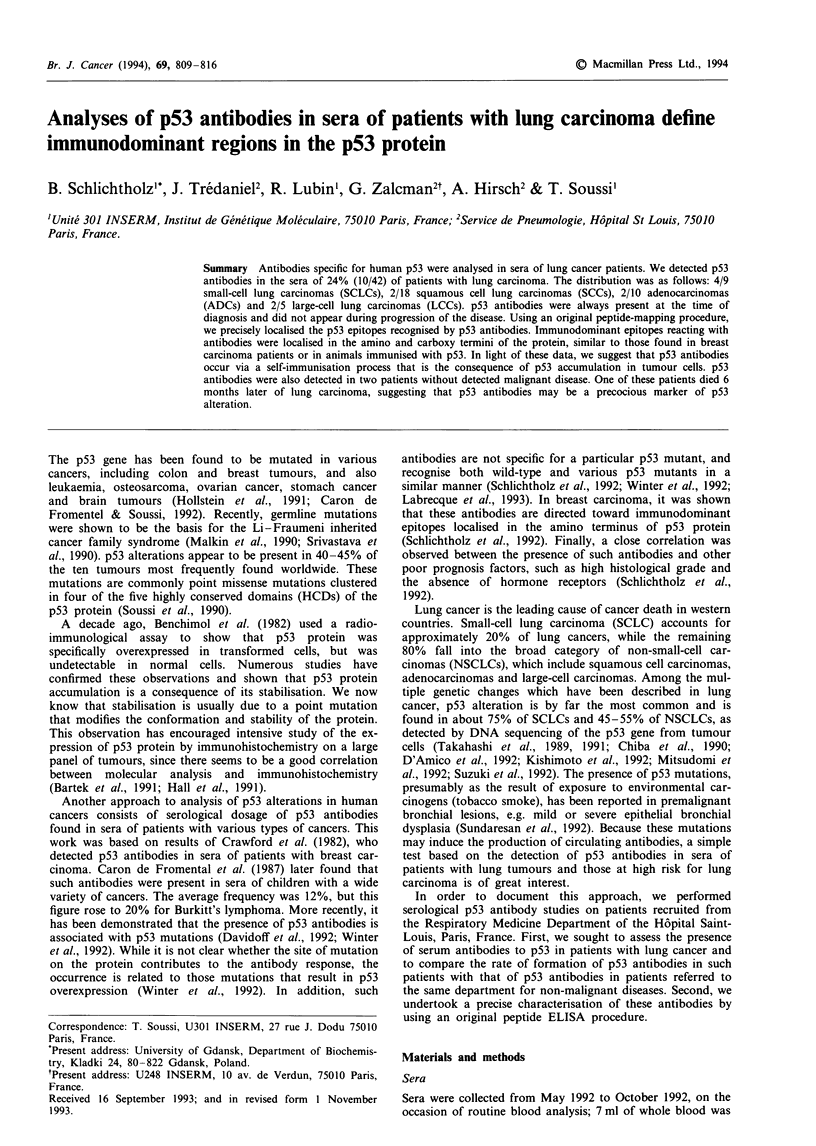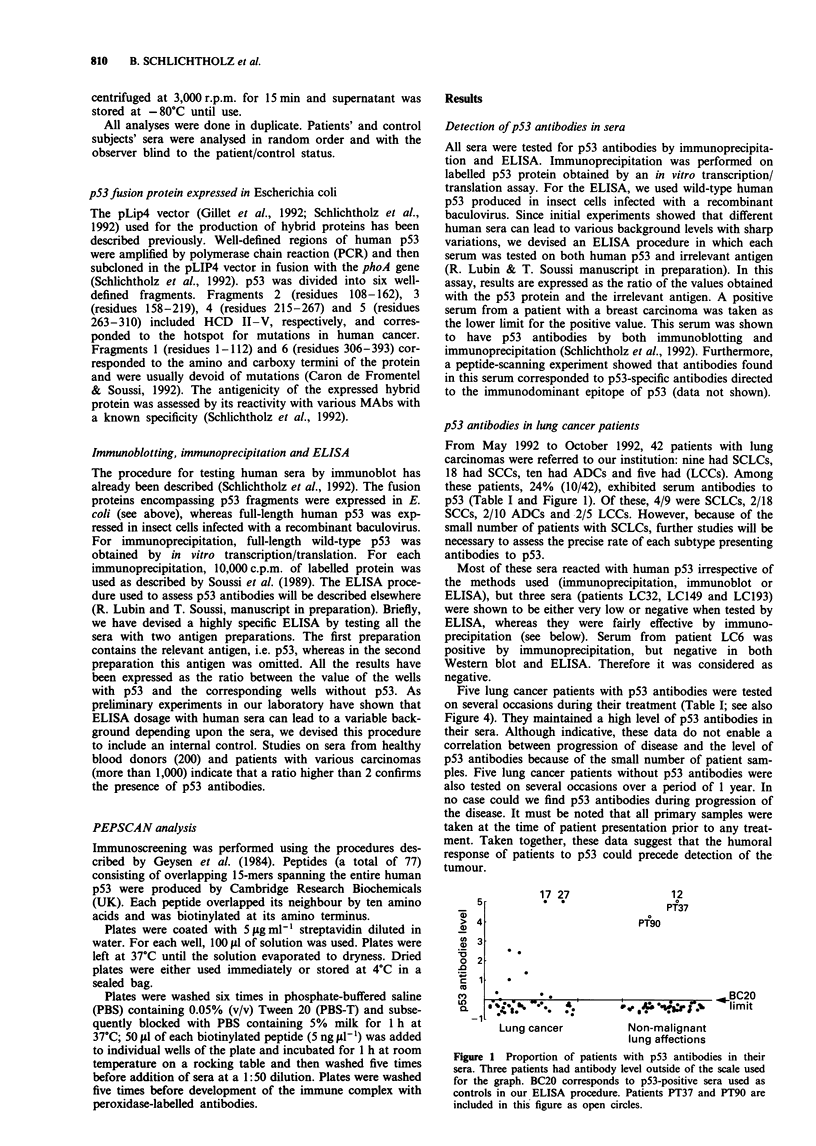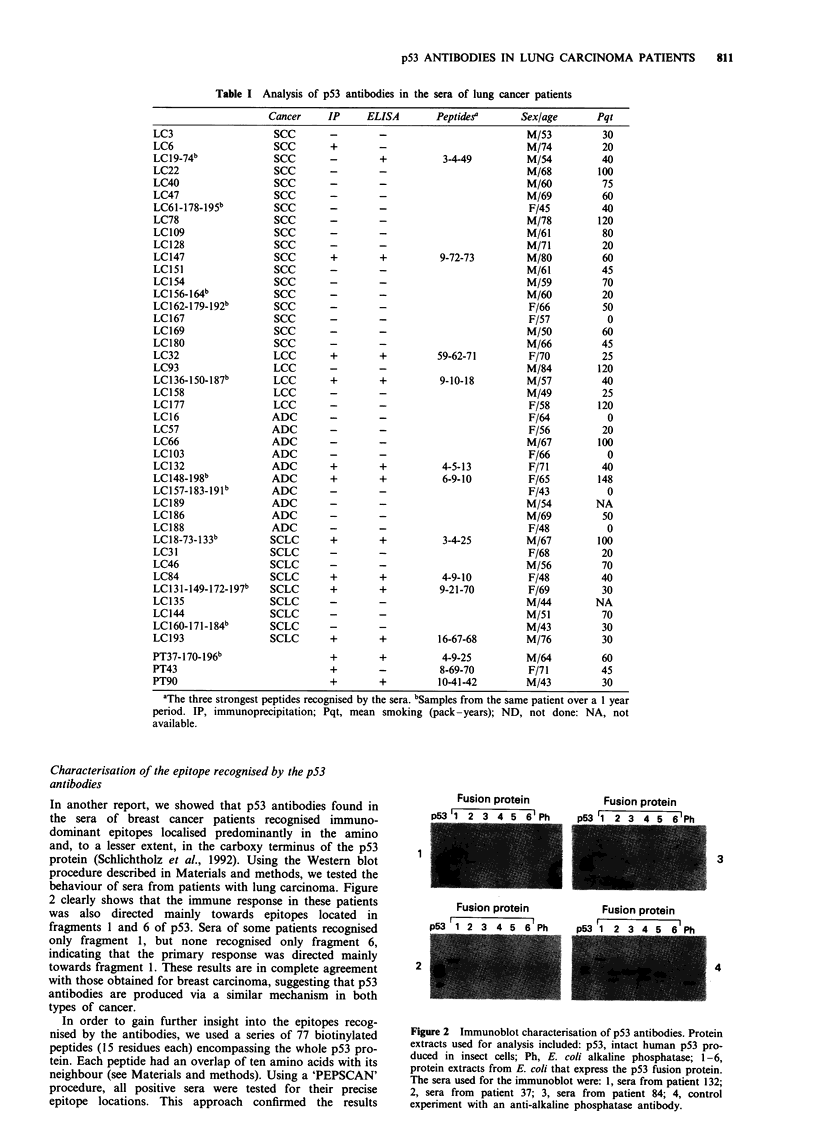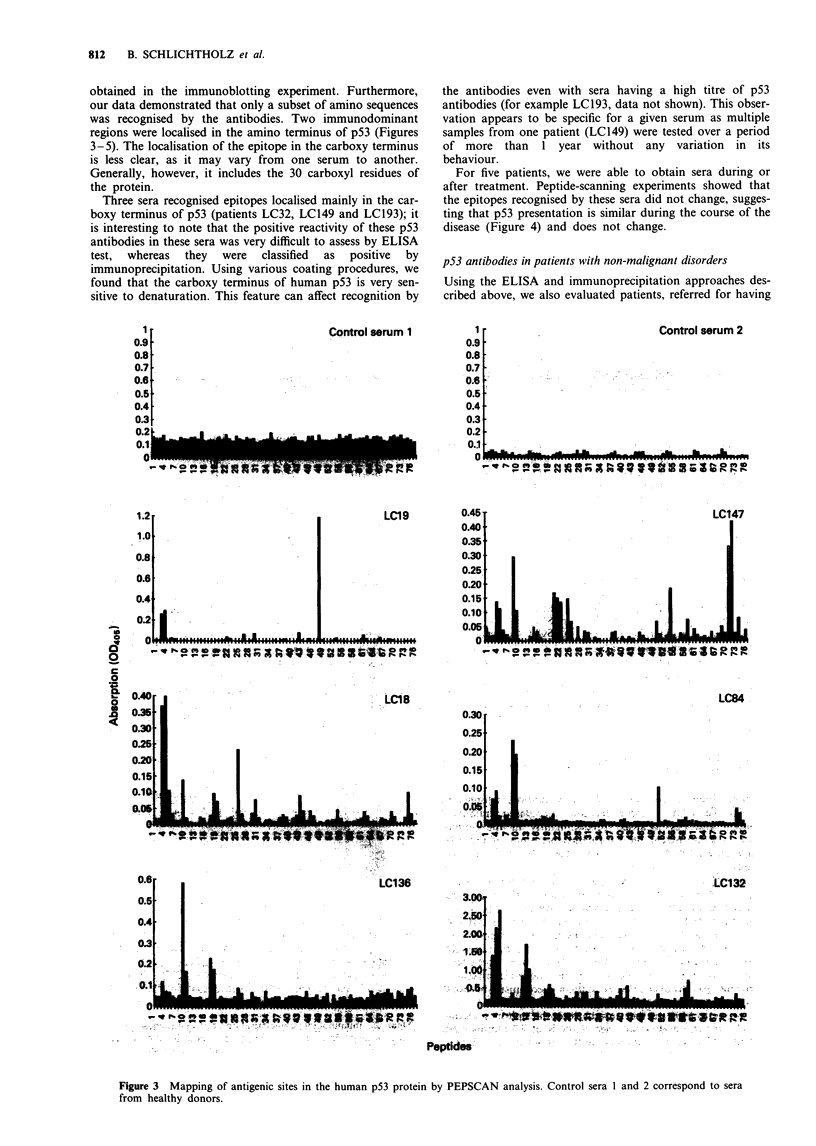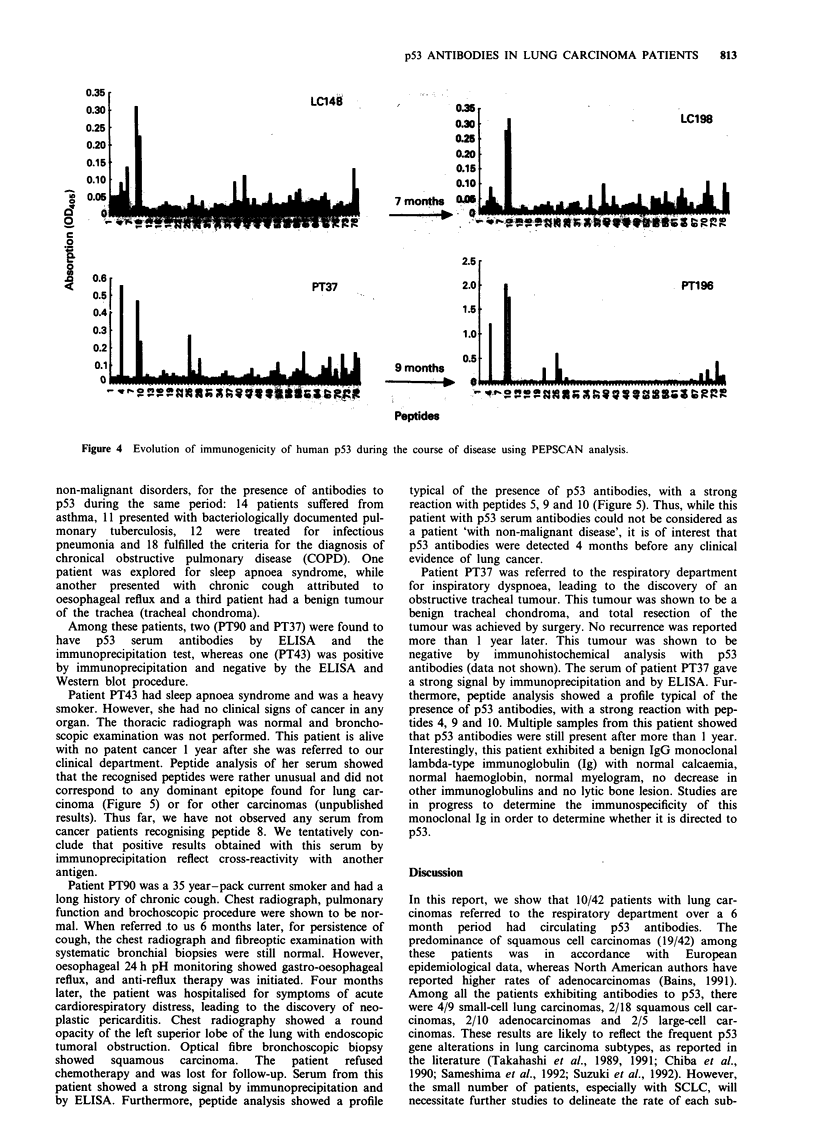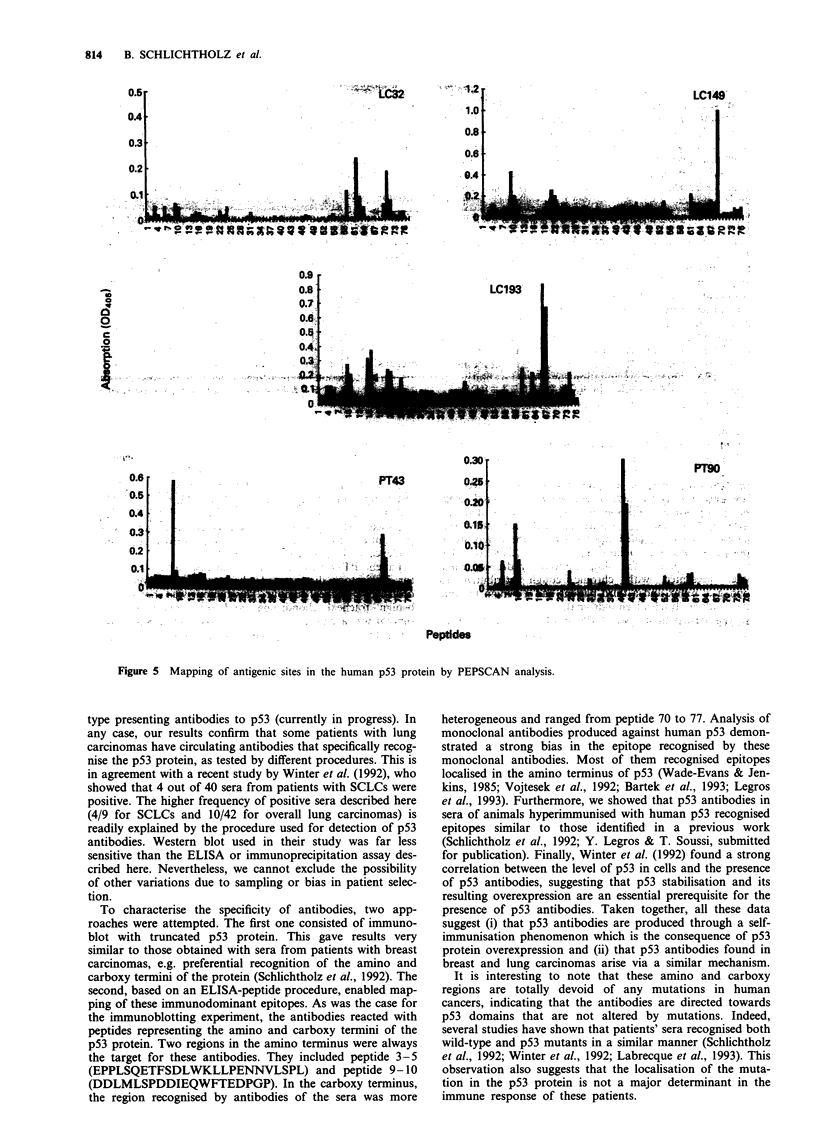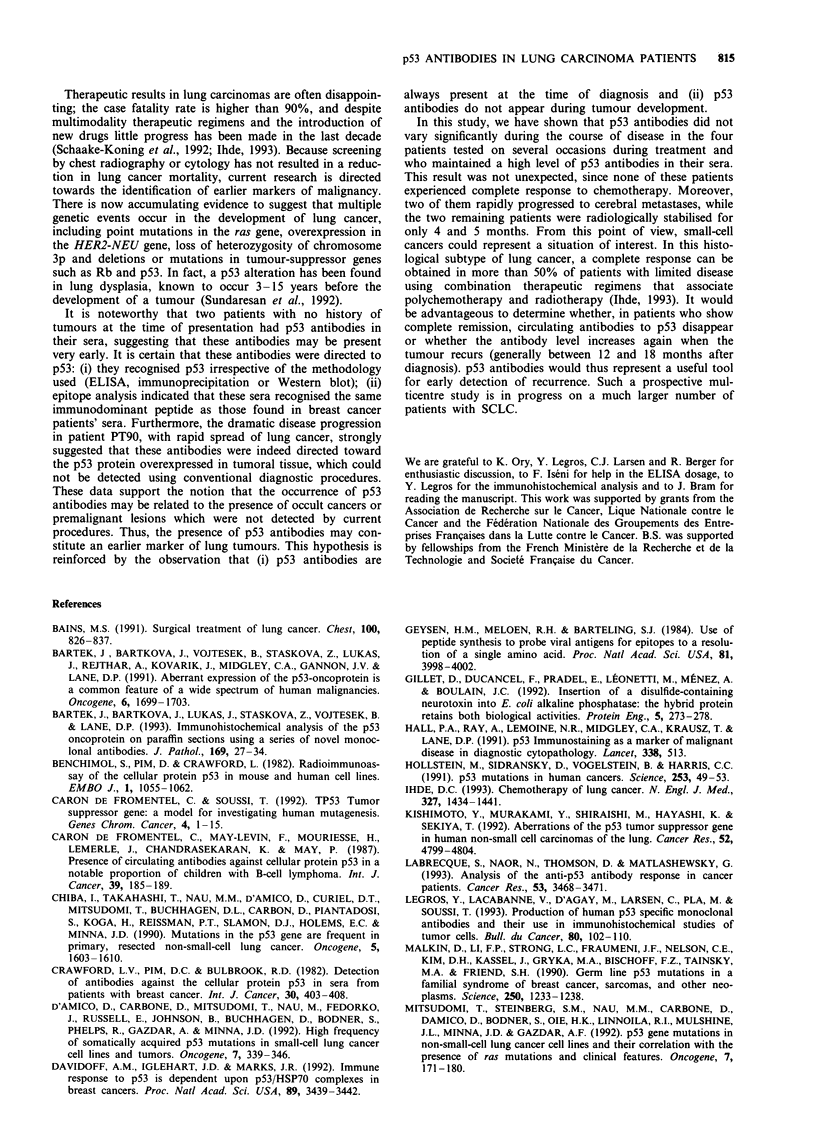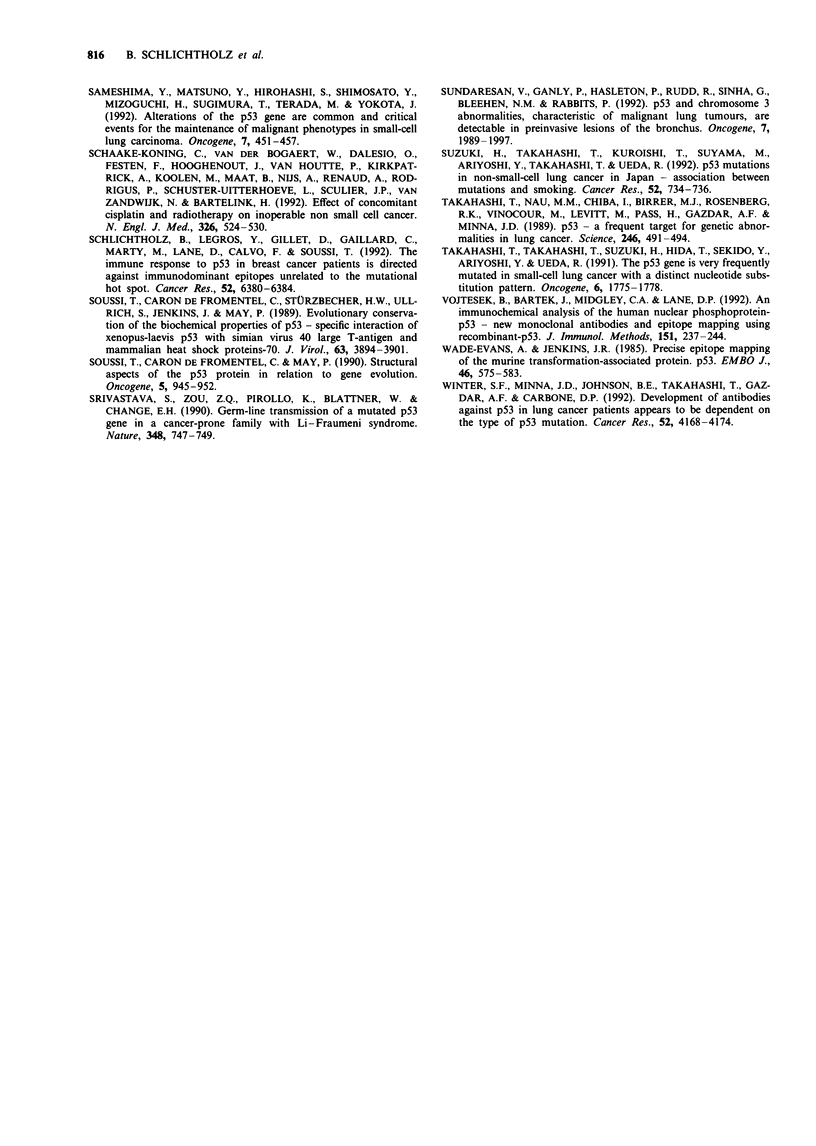# Analyses of p53 antibodies in sera of patients with lung carcinoma define immunodominant regions in the p53 protein.

**DOI:** 10.1038/bjc.1994.159

**Published:** 1994-05

**Authors:** B. Schlichtholz, J. Trédaniel, R. Lubin, G. Zalcman, A. Hirsch, T. Soussi

**Affiliations:** Unité 301 INSERM, Institut de Génétique Moléculaire, Paris, France.

## Abstract

**Images:**


Br. J. Cancer (1994), 69, 809-816 © Macmillan Press Ltd., 1994

### Analyses of p53 antibodies in sera of patients with lung carcinoma define immunodominant regions in the p53 protein

B. Schlichtholz[1*], J. Trédaniel[2], R. Lubin[1], G. Zalcman[2†], A. Hirsch[2] & T. Soussi[1]

[1]*Unité 301 INSERM, Institut de Génétique Moléculaire, 75010 Paris, France;* [2]*Service de Pneumologie, Hôpital St Louis, 75010 Paris, France.*

**Summary** Antibodies specific for human p53 were analysed in sera of lung cancer patients. We detected p53 antibodies in the sera of 24% (10/42) of patients with lung carcinoma. The distribution was as follows: 4/9 small-cell lung carcinomas (SCLCs), 2/18 squamous cell lung carcinomas (SCCs), 2/10 adenocarcinomas (ADCs) and 2/5 large-cell lung carcinomas (LCCs). p53 antibodies were always present at the time of diagnosis and did not appear during progression of the disease. Using an original peptide-mapping procedure, we precisely localised the p53 epitopes recognised by p53 antibodies. Immunodominant epitopes reacting with antibodies were localised in the amino and carboxy termini of the protein, similar to those found in breast carcinoma patients or in animals immunised with p53. In light of these data, we suggest that p53 antibodies occur via a self-immunisation process that is the consequence of p53 accumulation in tumour cells. p53 antibodies were also detected in two patients without detected malignant disease. One of these patients died 6 months later of lung carcinoma, suggesting that p53 antibodies may be a precocious marker of p53 alteration.

The p53 gene has been found to be mutated in various cancers, including colon and breast tumours, and also leukaemia, osteosarcoma, ovarian cancer, stomach cancer and brain tumours (Hollstein *et al.*, 1991; Caron de Fromentel & Soussi, 1992). Recently, germline mutations were shown to be the basis for the Li-Fraumeni inherited cancer family syndrome (Malkin *et al.*, 1990; Srivastava *et al.*, 1990). p53 alterations appear to be present in 40-45% of the ten tumours most frequently found worldwide. These mutations are commonly point missense mutations clustered in four of the five highly conserved domains (HCDs) of the p53 protein (Soussi *et al.*, 1990).

A decade ago, Benchimol *et al.* (1982) used a radio-immunological assay to show that p53 protein was specifically overexpressed in transformed cells, but was undetectable in normal cells. Numerous studies have confirmed these observations and shown that p53 protein accumulation is a consequence of its stabilisation. We now know that stabilisation is usually due to a point mutation that modifies the conformation and stability of the protein. This observation has encouraged intensive study of the expression of p53 protein by immunohistochemistry on a large panel of tumours, since there seems to be a good correlation between molecular analysis and immunohistochemistry (Bartek *et al.*, 1991; Hall *et al.*, 1991).

Another approach to analysis of p53 alterations in human cancers consists of serological dosage of p53 antibodies found in sera of patients with various types of cancers. This work was based on results of Crawford *et al.* (1982), who detected p53 antibodies in sera of patients with breast carcinoma. Caron de Fromental *et al.* (1987) later found that such antibodies were present in sera of children with a wide variety of cancers. The average frequency was 12%, but this figure rose to 20% for Burkitt's lymphoma. More recently, it has been demonstrated that the presence of p53 antibodies is associated with p53 mutations (Davidoff *et al.*, 1992; Winter *et al.*, 1992). While it is not clear whether the site of mutation on the protein contributes to the antibody response, the occurrence is related to those mutations that result in p53 overexpression (Winter *et al.*, 1992). In addition, such antibodies are not specific for a particular p53 mutant, and recognise both wild-type and various p53 mutants in a similar manner (Schlichtholz *et al.*, 1992; Winter *et al.*, 1992; Labrecque *et al.*, 1993). In breast carcinoma, it was shown that these antibodies are directed toward immunodominant epitopes localised in the amino terminus of p53 protein (Schlichtholz *et al.*, 1992). Finally, a close correlation was observed between the presence of such antibodies and other poor prognosis factors, such as high histological grade and the absence of hormone receptors (Schlichtholz *et al.*, 1992).

Lung cancer is the leading cause of cancer death in western countries. Small-cell lung carcinoma (SCLC) accounts for approximately 20% of lung cancers, while the remaining 80% fall into the broad category of non-small-cell carcinomas (NSCLCs), which include squamous cell carcinomas, adenocarcinomas and large-cell carcinomas. Among the multiple genetic changes which have been described in lung cancer, p53 alteration is by far the most common and is found in about 75% of SCLCs and 45-55% of NSCLCs, as detected by DNA sequencing of the p53 gene from tumour cells (Takahashi *et al.*, 1989, 1991; Chiba *et al.*, 1990; D'Amico *et al.*, 1992; Kishimoto *et al.*, 1992; Mitsudomi *et al.*, 1992; Suzuki *et al.*, 1992). The presence of p53 mutations, presumably as the result of exposure to environmental carcinogens (tobacco smoke), has been reported in premalignant bronchial lesions, e.g. mild or severe epithelial bronchial dysplasia (Sundaresan *et al.*, 1992). Because these mutations may induce the production of circulating antibodies, a simple test based on the detection of p53 antibodies in sera of patients with lung tumours and those at high risk for lung carcinoma is of great interest.

In order to document this approach, we performed serological p53 antibody studies on patients recruited from the Respiratory Medicine Department of the Hôpital Saint-Louis, Paris, France. First, we sought to assess the presence of serum antibodies to p53 in patients with lung cancer and to compare the rate of formation of p53 antibodies in such patients with that of p53 antibodies in patients referred to the same department for non-malignant diseases. Second, we undertook a precise characterisation of these antibodies by using an original peptide ELISA procedure.

### Materials and methods

### *Sera*

Sera were collected from May 1992 to October 1992, on the occasion of routine blood analysis; 7 ml of whole blood was

Correspondence: T. Soussi, U301 INSERM, 27 rue J. Dodu 75010 Paris, France.
*Present address: University of Gdansk, Department of Biochemistry, Kladki 24, 80-822 Gdansk, Poland.
†Present address: U248 INSERM, 10 av. de Verdun, 75010 Paris, France.
Received 16 September 1993; and in revised form 1 November 1993.

centrifuged at 3,000 r.p.m. for 15 min and supernatant was stored at $-80^{\circ}C$ until use.

All analyses were done in duplicate. Patients' and control subjects' sera were analysed in random order and with the observer blind to the patient/control status.

### *p53 fusion protein expressed in* Escherichia coli

The pLip4 vector (Gillet *et al.*, 1992; Schlichtholz *et al.*, 1992) used for the production of hybrid proteins has been described previously. Well-defined regions of human p53 were amplified by polymerase chain reaction (PCR) and then subcloned in the pLIP4 vector in fusion with the *phoA* gene (Schlichtholz *et al.*, 1992). p53 was divided into six well-defined fragments. Fragments 2 (residues 108–162), 3 (residues 158–219), 4 (residues 215–267) and 5 (residues 263–310) included HCD II–V, respectively, and corresponded to the hotspot for mutations in human cancer. Fragments 1 (residues 1–112) and 6 (residues 306–393) corresponded to the amino and carboxy termini of the protein and were usually devoid of mutations (Caron de Fromentel & Soussi, 1992). The antigenicity of the expressed hybrid protein was assessed by its reactivity with various MAbs with a known specificity (Schlichtholz *et al.*, 1992).

### *Immunoblotting, immunoprecipitation and ELISA*

The procedure for testing human sera by immunoblot has already been described (Schlichtholz *et al.*, 1992). The fusion proteins encompassing p53 fragments were expressed in *E. coli* (see above), whereas full-length human p53 was expressed in insect cells infected with a recombinant baculovirus. For immunoprecipitation, full-length wild-type p53 was obtained by *in vitro* transcription/translation. For each immunoprecipitation, 10,000 c.p.m. of labelled protein was used as described by Soussi *et al.* (1989). The ELISA procedure used to assess p53 antibodies will be described elsewhere (R. Lubin and T. Soussi, manuscript in preparation). Briefly, we have devised a highly specific ELISA by testing all the sera with two antigen preparations. The first preparation contains the relevant antigen, i.e. p53, whereas in the second preparation this antigen was omitted. All the results have been expressed as the ratio between the value of the wells with p53 and the corresponding wells without p53. As preliminary experiments in our laboratory have shown that ELISA dosage with human sera can lead to a variable background depending upon the sera, we devised this procedure to include an internal control. Studies on sera from healthy blood donors (200) and patients with various carcinomas (more than 1,000) indicate that a ratio higher than 2 confirms the presence of p53 antibodies.

### *PEPSCAN analysis*

Immunoscreening was performed using the procedures described by Geysen *et al.* (1984). Peptides (a total of 77) consisting of overlapping 15-mers spanning the entire human p53 were produced by Cambridge Research Biochemicals (UK). Each peptide overlapped its neighbour by ten amino acids and was biotinylated at its amino terminus.

Plates were coated with $5\,\mu g\,ml^{-1}$ streptavidin diluted in water. For each well, $100\,\mu l$ of solution was used. Plates were left at $37^{\circ}C$ until the solution evaporated to dryness. Dried plates were either used immediately or stored at $4^{\circ}C$ in a sealed bag.

Plates were washed six times in phosphate-buffered saline (PBS) containing 0.05% (v/v) Tween 20 (PBS-T) and subsequently blocked with PBS containing 5% milk for 1 h at $37^{\circ}C$; $50\,\mu l$ of each biotinylated peptide ($5\,ng\,\mu l^{-1}$) was added to individual wells of the plate and incubated for 1 h at room temperature on a rocking table and then washed five times before addition of sera at a 1:50 dilution. Plates were washed five times before development of the immune complex with peroxidase-labelled antibodies.

### Results

### *Detection of p53 antibodies in sera*

All sera were tested for p53 antibodies by immunoprecipitation and ELISA. Immunoprecipitation was performed on labelled p53 protein obtained by an *in vitro* transcription/translation assay. For the ELISA, we used wild-type human p53 produced in insect cells infected with a recombinant baculovirus. Since initial experiments showed that different human sera can lead to various background levels with sharp variations, we devised an ELISA procedure in which each serum was tested on both human p53 and irrelevant antigen (R. Lubin & T. Soussi manuscript in preparation). In this assay, results are expressed as the ratio of the values obtained with the p53 protein and the irrelevant antigen. A positive serum from a patient with a breast carcinoma was taken as the lower limit for the positive value. This serum was shown to have p53 antibodies by both immunoblotting and immunoprecipitation (Schlichtholz *et al.*, 1992). Furthermore, a peptide-scanning experiment showed that antibodies found in this serum corresponded to p53-specific antibodies directed to the immunodominant epitope of p53 (data not shown).

### *p53 antibodies in lung cancer patients*

From May 1992 to October 1992, 42 patients with lung carcinomas were referred to our institution: nine had SCLCs, 18 had SCCs, ten had ADCs and five had (LCCs). Among these patients, 24% (10/42), exhibited serum antibodies to p53 (Table I and Figure 1). Of these, 4/9 were SCLCs, 2/18 SCCs, 2/10 ADCs and 2/5 LCCs. However, because of the small number of patients with SCLCs, further studies will be necessary to assess the precise rate of each subtype presenting antibodies to p53.

Most of these sera reacted with human p53 irrespective of the methods used (immunoprecipitation, immunoblot or ELISA), but three sera (patients LC32, LC149 and LC193) were shown to be either very low or negative when tested by ELISA, whereas they were fairly effective by immunoprecipitation (see below). Serum from patient LC6 was positive by immunoprecipitation, but negative in both Western blot and ELISA. Therefore it was considered as negative.

Five lung cancer patients with p53 antibodies were tested on several occasions during their treatment (Table I; see also Figure 4). They maintained a high level of p53 antibodies in their sera. Although indicative, these data do not enable a correlation between progression of disease and the level of p53 antibodies because of the small number of patient samples. Five lung cancer patients without p53 antibodies were also tested on several occasions over a period of 1 year. In no case could we find p53 antibodies during progression of the disease. It must be noted that all primary samples were taken at the time of patient presentation prior to any treatment. Taken together, these data suggest that the humoral response of patients to p53 could precede detection of the tumour.

**Figure 1** Proportion of patients with p53 antibodies in their sera. Three patients had antibody level outside of the scale used for the graph. BC20 corresponds to p53-positive sera used as controls in our ELISA procedure. Patients PT37 and PT90 are included in this figure as open circles.

**Table I** Analysis of p53 antibodies in the sera of lung cancer patients

| | *Cancer* | *IP* | *ELISA* | *Peptides*[a] | *Sex/age* | *Pqt* |
|---|---|---|---|---|---|---|
| LC3 | SCC | – | – | | M/53 | 30 |
| LC6 | SCC | + | – | | M/74 | 20 |
| LC19-74[b] | SCC | – | + | 3-4-49 | M/54 | 40 |
| LC22 | SCC | – | – | | M/68 | 100 |
| LC40 | SCC | – | – | | M/60 | 75 |
| LC47 | SCC | – | – | | M/69 | 60 |
| LC61-178-195[b] | SCC | – | – | | F/45 | 40 |
| LC78 | SCC | – | – | | M/78 | 120 |
| LC109 | SCC | – | – | | M/61 | 80 |
| LC128 | SCC | – | – | | M/71 | 20 |
| LC147 | SCC | + | + | 9-72-73 | M/80 | 60 |
| LC151 | SCC | – | – | | M/61 | 45 |
| LC154 | SCC | – | – | | M/59 | 70 |
| LC156-164[b] | SCC | – | – | | M/60 | 20 |
| LC162-179-192[b] | SCC | – | – | | F/66 | 50 |
| LC167 | SCC | – | – | | F/57 | 0 |
| LC169 | SCC | – | – | | M/50 | 60 |
| LC180 | SCC | – | – | | M/66 | 45 |
| LC32 | LCC | + | + | 59-62-71 | F/70 | 25 |
| LC93 | LCC | – | – | | M/84 | 120 |
| LC136-150-187[b] | LCC | + | + | 9-10-18 | M/57 | 40 |
| LC158 | LCC | – | – | | M/49 | 25 |
| LC177 | LCC | – | – | | F/58 | 120 |
| LC16 | ADC | – | – | | F/64 | 0 |
| LC57 | ADC | – | – | | F/56 | 20 |
| LC66 | ADC | – | – | | M/67 | 100 |
| LC103 | ADC | – | – | | F/66 | 0 |
| LC132 | ADC | + | + | 4-5-13 | F/71 | 40 |
| LC148-198[b] | ADC | + | + | 6-9-10 | F/65 | 148 |
| LC157-183-191[b] | ADC | – | – | | F/43 | 0 |
| LC189 | ADC | – | – | | M/54 | NA |
| LC186 | ADC | – | – | | M/69 | 50 |
| LC188 | ADC | – | – | | F/48 | 0 |
| LC18-73-133[b] | SCLC | + | + | 3-4-25 | M/67 | 100 |
| LC31 | SCLC | – | – | | F/68 | 20 |
| LC46 | SCLC | – | – | | M/56 | 70 |
| LC84 | SCLC | + | + | 4-9-10 | F/48 | 40 |
| LC131-149-172-197[b] | SCLC | + | + | 9-21-70 | F/69 | 30 |
| LC135 | SCLC | – | – | | M/44 | NA |
| LC144 | SCLC | – | – | | M/51 | 70 |
| LC160-171-184[b] | SCLC | – | – | | M/43 | 30 |
| LC193 | SCLC | + | + | 16-67-68 | M/76 | 30 |
| PT37-170-196[b] | | + | + | 4-9-25 | M/64 | 60 |
| PT43 | | + | – | 8-69-70 | F/71 | 45 |
| PT90 | | + | + | 10-41-42 | M/43 | 30 |

[a]The three strongest peptides recognised by the sera. [b]Samples from the same patient over a 1 year period. IP, immunoprecipitation; Pqt, mean smoking (pack-years); ND, not done: NA, not available.

### *Characterisation of the epitope recognised by the p53 antibodies*

In another report, we showed that p53 antibodies found in the sera of breast cancer patients recognised immunodominant epitopes localised predominantly in the amino and, to a lesser extent, in the carboxy terminus of the p53 protein (Schlichtholz *et al.*, 1992). Using the Western blot procedure described in Materials and methods, we tested the behaviour of sera from patients with lung carcinoma. Figure 2 clearly shows that the immune response in these patients was also directed mainly towards epitopes located in fragments 1 and 6 of p53. Sera of some patients recognised only fragment 1, but none recognised only fragment 6, indicating that the primary response was directed mainly towards fragment 1. These results are in complete agreement with those obtained for breast carcinoma, suggesting that p53 antibodies are produced via a similar mechanism in both types of cancer.

In order to gain further insight into the epitopes recognised by the antibodies, we used a series of 77 biotinylated peptides (15 residues each) encompassing the whole p53 protein. Each peptide had an overlap of ten amino acids with its neighbour (see Materials and methods). Using a 'PEPSCAN' procedure, all positive sera were tested for their precise epitope locations. This approach confirmed the results

**Figure 2** Immunoblot characterisation of p53 antibodies. Protein extracts used for analysis included: p53, intact human p53 produced in insect cells; Ph, *E. coli* alkaline phosphatase; 1–6, protein extracts from *E. coli* that express the p53 fusion protein. The sera used for the immunoblot were: 1, sera from patient 132; 2, sera from patient 37; 3, sera from patient 84; 4, control experiment with an anti-alkaline phosphatase antibody.

obtained in the immunoblotting experiment. Furthermore, our data demonstrated that only a subset of amino sequences was recognised by the antibodies. Two immunodominant regions were localised in the amino terminus of p53 (Figures 3–5). The localisation of the epitope in the carboxy terminus is less clear, as it may vary from one serum to another. Generally, however, it includes the 30 carboxyl residues of the protein.

Three sera recognised epitopes localised mainly in the carboxy terminus of p53 (patients LC32, LC149 and LC193); it is interesting to note that the positive reactivity of these p53 antibodies in these sera was very difficult to assess by ELISA test, whereas they were classified as positive by immunoprecipitation. Using various coating procedures, we found that the carboxy terminus of human p53 is very sensitive to denaturation. This feature can affect recognition by the antibodies even with sera having a high titre of p53 antibodies (for example LC193, data not shown). This observation appears to be specific for a given serum as multiple samples from one patient (LC149) were tested over a period of more than 1 year without any variation in its behaviour.

For five patients, we were able to obtain sera during or after treatment. Peptide-scanning experiments showed that the epitopes recognised by these sera did not change, suggesting that p53 presentation is similar during the course of the disease (Figure 4) and does not change.

*p53 antibodies in patients with non-malignant disorders*

Using the ELISA and immunoprecipitation approaches described above, we also evaluated patients, referred for having

**Figure 3** Mapping of antigenic sites in the human p53 protein by PEPSCAN analysis. Control sera 1 and 2 correspond to sera from healthy donors.

**Figure 4** Evolution of immunogenicity of human p53 during the course of disease using PEPSCAN analysis.

non-malignant disorders, for the presence of antibodies to p53 during the same period: 14 patients suffered from asthma, 11 presented with bacteriologically documented pulmonary tuberculosis, 12 were treated for infectious pneumonia and 18 fulfilled the criteria for the diagnosis of chronical obstructive pulmonary disease (COPD). One patient was explored for sleep apnoea syndrome, while another presented with chronic cough attributed to oesophageal reflux and a third patient had a benign tumour of the trachea (tracheal chondroma).

Among these patients, two (PT90 and PT37) were found to have p53 serum antibodies by ELISA and the immunoprecipitation test, whereas one (PT43) was positive by immunoprecipitation and negative by the ELISA and Western blot procedure.

Patient PT43 had sleep apnoea syndrome and was a heavy smoker. However, she had no clinical signs of cancer in any organ. The thoracic radiograph was normal and bronchoscopic examination was not performed. This patient is alive with no patent cancer 1 year after she was referred to our clinical department. Peptide analysis of her serum showed that the recognised peptides were rather unusual and did not correspond to any dominant epitope found for lung carcinoma (Figure 5) or for other carcinomas (unpublished results). Thus far, we have not observed any serum from cancer patients recognising peptide 8. We tentatively conclude that positive results obtained with this serum by immunoprecipitation reflect cross-reactivity with another antigen.

Patient PT90 was a 35 year–pack current smoker and had a long history of chronic cough. Chest radiograph, pulmonary function and brochoscopic procedure were shown to be normal. When referred to us 6 months later, for persistence of cough, the chest radiograph and fibreoptic examination with systematic bronchial biopsies were still normal. However, oesophageal 24 h pH monitoring showed gastro-oesophageal reflux, and anti-reflux therapy was initiated. Four months later, the patient was hospitalised for symptoms of acute cardiorespiratory distress, leading to the discovery of neoplastic pericarditis. Chest radiography showed a round opacity of the left superior lobe of the lung with endoscopic tumoral obstruction. Optical fibre bronchoscopic biopsy showed squamous carcinoma. The patient refused chemotherapy and was lost for follow-up. Serum from this patient showed a strong signal by immunoprecipitation and by ELISA. Furthermore, peptide analysis showed a profile typical of the presence of p53 antibodies, with a strong reaction with peptides 5, 9 and 10 (Figure 5). Thus, while this patient with p53 serum antibodies could not be considered as a patient 'with non-malignant disease', it is of interest that p53 antibodies were detected 4 months before any clinical evidence of lung cancer.

Patient PT37 was referred to the respiratory department for inspiratory dyspnoea, leading to the discovery of an obstructive tracheal tumour. This tumour was shown to be a benign tracheal chondroma, and total resection of the tumour was achieved by surgery. No recurrence was reported more than 1 year later. This tumour was shown to be negative by immunohistochemical analysis with p53 antibodies (data not shown). The serum of patient PT37 gave a strong signal by immunoprecipitation and by ELISA. Furthermore, peptide analysis showed a profile typical of the presence of p53 antibodies, with a strong reaction with peptides 4, 9 and 10. Multiple samples from this patient showed that p53 antibodies were still present after more than 1 year. Interestingly, this patient exhibited a benign IgG monoclonal lambda-type immunoglobulin (Ig) with normal calcaemia, normal haemoglobin, normal myelogram, no decrease in other immunoglobulins and no lytic bone lesion. Studies are in progress to determine the immunospecificity of this monoclonal Ig in order to determine whether it is directed to p53.

### Discussion

In this report, we show that 10/42 patients with lung carcinomas referred to the respiratory department over a 6 month period had circulating p53 antibodies. The predominance of squamous cell carcinomas (19/42) among these patients was in accordance with European epidemiological data, whereas North American authors have reported higher rates of adenocarcinomas (Bains, 1991). Among all the patients exhibiting antibodies to p53, there were 4/9 small-cell lung carcinomas, 2/18 squamous cell carcinomas, 2/10 adenocarcinomas and 2/5 large-cell carcinomas. These results are likely to reflect the frequent p53 gene alterations in lung carcinoma subtypes, as reported in the literature (Takahashi *et al.*, 1989, 1991; Chiba *et al.*, 1990; Sameshima *et al.*, 1992; Suzuki *et al.*, 1992). However, the small number of patients, especially with SCLC, will necessitate further studies to delineate the rate of each sub-

**Figure 5** Mapping of antigenic sites in the human p53 protein by PEPSCAN analysis.

type presenting antibodies to p53 (currently in progress). In any case, our results confirm that some patients with lung carcinomas have circulating antibodies that specifically recognise the p53 protein, as tested by different procedures. This is in agreement with a recent study by Winter *et al.* (1992), who showed that 4 out of 40 sera from patients with SCLCs were positive. The higher frequency of positive sera described here (4/9 for SCLCs and 10/42 for overall lung carcinomas) is readily explained by the procedure used for detection of p53 antibodies. Western blot used in their study was far less sensitive than the ELISA or immunoprecipitation assay described here. Nevertheless, we cannot exclude the possibility of other variations due to sampling or bias in patient selection.

To characterise the specificity of antibodies, two approaches were attempted. The first one consisted of immunoblot with truncated p53 protein. This gave results very similar to those obtained with sera from patients with breast carcinomas, e.g. preferential recognition of the amino and carboxy termini of the protein (Schlichtholz *et al.*, 1992). The second, based on an ELISA-peptide procedure, enabled mapping of these immunodominant epitopes. As was the case for the immunoblotting experiment, the antibodies reacted with peptides representing the amino and carboxy termini of the p53 protein. Two regions in the amino terminus were always the target for these antibodies. They included peptide 3–5 (EPPLSQETFSDLWKLLPENNVLSPL) and peptide 9–10 (DDLMLSPDDIEQWFTEDPGP). In the carboxy terminus, the region recognised by antibodies of the sera was more heterogeneous and ranged from peptide 70 to 77. Analysis of monoclonal antibodies produced against human p53 demonstrated a strong bias in the epitope recognised by these monoclonal antibodies. Most of them recognised epitopes localised in the amino terminus of p53 (Wade-Evans & Jenkins, 1985; Vojtesek *et al.*, 1992; Bartek *et al.*, 1993; Legros *et al.*, 1993). Furthermore, we showed that p53 antibodies in sera of animals hyperimmunised with human p53 recognised epitopes similar to those identified in a previous work (Schlichtholz *et al.*, 1992; Y. Legros & T. Soussi, submitted for publication). Finally, Winter *et al.* (1992) found a strong correlation between the level of p53 in cells and the presence of p53 antibodies, suggesting that p53 stabilisation and its resulting overexpression are an essential prerequisite for the presence of p53 antibodies. Taken together, all these data suggest (i) that p53 antibodies are produced through a self-immunisation phenomenon which is the consequence of p53 protein overexpression and (ii) that p53 antibodies found in breast and lung carcinomas arise via a similar mechanism.

It is interesting to note that these amino and carboxy regions are totally devoid of any mutations in human cancers, indicating that the antibodies are directed towards p53 domains that are not altered by mutations. Indeed, several studies have shown that patients' sera recognised both wild-type and p53 mutants in a similar manner (Schlichtholz *et al.*, 1992; Winter *et al.*, 1992; Labrecque *et al.*, 1993). This observation also suggests that the localisation of the mutation in the p53 protein is not a major determinant in the immune response of these patients.

Therapeutic results in lung carcinomas are often disappointing; the case fatality rate is higher than 90%, and despite multimodality therapeutic regimens and the introduction of new drugs little progress has been made in the last decade (Schaake-Koning *et al.*, 1992; Ihde, 1993). Because screening by chest radiography or cytology has not resulted in a reduction in lung cancer mortality, current research is directed towards the identification of earlier markers of malignancy. There is now accumulating evidence to suggest that multiple genetic events occur in the development of lung cancer, including point mutations in the *ras* gene, overexpression in the *HER2-NEU* gene, loss of heterozygosity of chromosome 3p and deletions or mutations in tumour-suppressor genes such as Rb and p53. In fact, a p53 alteration has been found in lung dysplasia, known to occur 3–15 years before the development of a tumour (Sundaresan *et al.*, 1992).

It is noteworthy that two patients with no history of tumours at the time of presentation had p53 antibodies in their sera, suggesting that these antibodies may be present very early. It is certain that these antibodies were directed to p53: (i) they recognised p53 irrespective of the methodology used (ELISA, immunoprecipitation or Western blot); (ii) epitope analysis indicated that these sera recognised the same immunodominant peptide as those found in breast cancer patients' sera. Furthermore, the dramatic disease progression in patient PT90, with rapid spread of lung cancer, strongly suggested that these antibodies were indeed directed toward the p53 protein overexpressed in tumoral tissue, which could not be detected using conventional diagnostic procedures. These data support the notion that the occurrence of p53 antibodies may be related to the presence of occult cancers or premalignant lesions which were not detected by current procedures. Thus, the presence of p53 antibodies may constitute an earlier marker of lung tumours. This hypothesis is reinforced by the observation that (i) p53 antibodies are always present at the time of diagnosis and (ii) p53 antibodies do not appear during tumour development.

In this study, we have shown that p53 antibodies did not vary significantly during the course of disease in the four patients tested on several occasions during treatment and who maintained a high level of p53 antibodies in their sera. This result was not unexpected, since none of these patients experienced complete response to chemotherapy. Moreover, two of them rapidly progressed to cerebral metastases, while the two remaining patients were radiologically stabilised for only 4 and 5 months. From this point of view, small-cell cancers could represent a situation of interest. In this histological subtype of lung cancer, a complete response can be obtained in more than 50% of patients with limited disease using combination therapeutic regimens that associate polychemotherapy and radiotherapy (Ihde, 1993). It would be advantageous to determine whether, in patients who show complete remission, circulating antibodies to p53 disappear or whether the antibody level increases again when the tumour recurs (generally between 12 and 18 months after diagnosis). p53 antibodies would thus represent a useful tool for early detection of recurrence. Such a prospective multicentre study is in progress on a much larger number of patients with SCLC.

We are grateful to K. Ory, Y. Legros, C.J. Larsen and R. Berger for enthusiastic discussion, to F. Iséni for help in the ELISA dosage, to Y. Legros for the immunohistochemical analysis and to J. Bram for reading the manuscript. This work was supported by grants from the Association de Recherche sur le Cancer, Lique Nationale contre le Cancer and the Fédération Nationale des Groupements des Entreprises Françaises dans la Lutte contre le Cancer. B.S. was supported by fellowships from the French Ministère de la Recherche et de la Technologie and Societé Française du Cancer.

### References

BAINS, M.S. (1991). Surgical treatment of lung cancer. *Chest*, **100**, 826–837.

BARTEK, J , BARTKOVA, J., VOJTESEK, B., STASKOVA, Z., LUKAS, J., REJTHAR, A., KOVARIK, J., MIDGLEY, C.A., GANNON, J.V. & LANE, D.P. (1991). Aberrant expression of the p53-oncoprotein is a common feature of a wide spectrum of human malignancies. *Oncogene*, **6**, 1699–1703.

BARTEK, J., BARTKOVA, J., LUKAS, J., STASKOVA, Z., VOJTESEK, B. & LANE, D.P. (1993). Immunohistochemical analysis of the p53 oncoprotein on paraffin sections using a series of novel monoclonal antibodies. *J. Pathol.*, **169**, 27–34.

BENCHIMOL, S., PIM, D. & CRAWFORD, L. (1982). Radioimmunoassay of the cellular protein p53 in mouse and human cell lines. *EMBO J.*, **1**, 1055–1062.

CARON DE FROMENTEL, C. & SOUSSI, T. (1992). TP53 Tumor suppressor gene: a model for investigating human mutagenesis. *Genes Chrom. Cancer*, **4**, 1–15.

CARON DE FROMENTEL, C., MAY-LEVIN, F., MOURIESSE, H., LEMERLE, J., CHANDRASEKARAN, K. & MAY, P. (1987). Presence of circulating antibodies against cellular protein p53 in a notable proportion of children with B-cell lymphoma. *Int. J. Cancer*, **39**, 185–189.

CHIBA, I., TAKAHASHI, T., NAU, M.M., D'AMICO, D., CURIEL, D.T., MITSUDOMI, T., BUCHHAGEN, D.L., CARBON, D., PIANTADOSI, S., KOGA, H., REISSMAN, P.T., SLAMON, D.J., HOLEMS, E.C. & MINNA, J.D. (1990). Mutations in the p53 gene are frequent in primary, resected non-small-cell lung cancer. *Oncogene*, **5**, 1603–1610.

CRAWFORD, L.V., PIM, D.C. & BULBROOK, R.D. (1982). Detection of antibodies against the cellular protein p53 in sera from patients with breast cancer. *Int. J. Cancer*, **30**, 403–408.

D'AMICO, D., CARBONE, D., MITSUDOMI, T., NAU, M., FEDORKO, J., RUSSELL, E., JOHNSON, B., BUCHHAGEN, D., BODNER, S., PHELPS, R., GAZDAR, A. & MINNA, J.D. (1992). High frequency of somatically acquired p53 mutations in small-cell lung cancer cell lines and tumors. *Oncogene*, **7**, 339–346.

DAVIDOFF, A.M., IGLEHART, J.D. & MARKS, J.R. (1992). Immune response to p53 is dependent upon p53/HSP70 complexes in breast cancers. *Proc. Natl Acad. Sci. USA*, **89**, 3439–3442.

GEYSEN, H.M., MELOEN, R.H. & BARTELING, S.J. (1984). Use of peptide synthesis to probe viral antigens for epitopes to a resolution of a single amino acid. *Proc. Natl Acad. Sci. USA*, **81**, 3998–4002.

GILLET, D., DUCANCEL, F., PRADEL, E., LÉONETTI, M., MÉNEZ, A. & BOULAIN, J.C. (1992). Insertion of a disulfide-containing neurotoxin into *E. coli* alkaline phosphatase: the hybrid protein retains both biological activities. *Protein Eng.*, **5**, 273–278.

HALL, P.A., RAY, A., LEMOINE, N.R., MIDGLEY, C.A., KRAUSZ, T. & LANE, D.P. (1991). p53 Immunostaining as a marker of malignant disease in diagnostic cytopathology. *Lancet*, **338**, 513.

HOLLSTEIN, M., SIDRANSKY, D., VOGELSTEIN, B. & HARRIS, C.C. (1991). p53 mutations in human cancers. *Science*, **253**, 49–53.

IHDE, D.C. (1993). Chemotherapy of lung cancer. *N. Engl. J. Med.*, **327**, 1434–1441.

KISHIMOTO, Y., MURAKAMI, Y., SHIRAISHI, M., HAYASHI, K. & SEKIYA, T. (1992). Aberrations of the p53 tumor suppressor gene in human non-small cell carcinomas of the lung. *Cancer Res.*, **52**, 4799–4804.

LABRECQUE, S., NAOR, N., THOMSON, D. & MATLASHEWSKY, G. (1993). Analysis of the anti-p53 antibody response in cancer patients. *Cancer Res.*, **53**, 3468–3471.

LEGROS, Y., LACABANNE, V., D'AGAY, M., LARSEN, C., PLA, M. & SOUSSI, T. (1993). Production of human p53 specific monoclonal antibodies and their use in immunohistochemical studies of tumor cells. *Bull. du Cancer*, **80**, 102–110.

MALKIN, D., LI, F.P., STRONG, L.C., FRAUMENI, J.F., NELSON, C.E., KIM, D.H., KASSEL, J., GRYKA, M.A., BISCHOFF, F.Z., TAINSKY, M.A. & FRIEND, S.H. (1990). Germ line p53 mutations in a familial syndrome of breast cancer, sarcomas, and other neoplasms. *Science*, **250**, 1233–1238.

MITSUDOMI, T., STEINBERG, S.M., NAU, M.M., CARBONE, D., DAMICO, D., BODNER, S., OIE, H.K., LINNOILA, R.I., MULSHINE, J.L., MINNA, J.D. & GAZDAR, A.F. (1992). p53 gene mutations in non-small-cell lung cancer cell lines and their correlation with the presence of *ras* mutations and clinical features. *Oncogene*, **7**, 171–180.

SAMESHIMA, Y., MATSUNO, Y., HIROHASHI, S., SHIMOSATO, Y., MIZOGUCHI, H., SUGIMURA, T., TERADA, M. & YOKOTA, J. (1992). Alterations of the p53 gene are common and critical events for the maintenance of malignant phenotypes in small-cell lung carcinoma. *Oncogene*, **7**, 451-457.

SCHAAKE-KONING, C., VAN DER BOGAERT, W., DALESIO, O., FESTEN, F., HOOGHENOUT, J., VAN HOUTTE, P., KIRKPATRICK, A., KOOLEN, M., MAAT, B., NIJS, A., RENAUD, A., RODRIGUS, P., SCHUSTER-UITTERHOEVE, L., SCULIER, J.P., VAN ZANDWIJK, N. & BARTELINK, H. (1992). Effect of concomitant cisplatin and radiotherapy on inoperable non small cell cancer. *N. Engl. J. Med.*, **326**, 524-530.

SCHLICHTHOLZ, B., LEGROS, Y., GILLET, D., GAILLARD, C., MARTY, M., LANE, D., CALVO, F. & SOUSSI, T. (1992). The immune response to p53 in breast cancer patients is directed against immunodominant epitopes unrelated to the mutational hot spot. *Cancer Res.*, **52**, 6380-6384.

SOUSSI, T., CARON DE FROMENTEL, C., STÜRZBECHER, H.W., ULLRICH, S., JENKINS, J. & MAY, P. (1989). Evolutionary conservation of the biochemical properties of p53 - specific interaction of xenopus-laevis p53 with simian virus 40 large T-antigen and mammalian heat shock proteins-70. *J. Virol.*, **63**, 3894-3901.

SOUSSI, T., CARON DE FROMENTEL, C. & MAY, P. (1990). Structural aspects of the p53 protein in relation to gene evolution. *Oncogene*, **5**, 945-952.

SRIVASTAVA, S., ZOU, Z.Q., PIROLLO, K., BLATTNER, W. & CHANGE, E.H. (1990). Germ-line transmission of a mutated p53 gene in a cancer-prone family with Li-Fraumeni syndrome. *Nature*, **348**, 747-749.

SUNDARESAN, V., GANLY, P., HASLETON, P., RUDD, R., SINHA, G., BLEEHEN, N.M. & RABBITS, P. (1992). p53 and chromosome 3 abnormalities, characteristic of malignant lung tumours, are detectable in preinvasive lesions of the bronchus. *Oncogene*, **7**, 1989-1997.

SUZUKI, H., TAKAHASHI, T., KUROISHI, T., SUYAMA, M., ARIYOSHI, Y., TAKAHASHI, T. & UEDA, R. (1992). p53 mutations in non-small-cell lung cancer in Japan - association between mutations and smoking. *Cancer Res.*, **52**, 734-736.

TAKAHASHI, T., NAU, M.M., CHIBA, I., BIRRER, M.J., ROSENBERG, R.K., VINOCOUR, M., LEVITT, M., PASS, H., GAZDAR, A.F. & MINNA, J.D. (1989). p53 - a frequent target for genetic abnormalities in lung cancer. *Science*, **246**, 491-494.

TAKAHASHI, T., TAKAHASHI, T., SUZUKI, H., HIDA, T., SEKIDO, Y., ARIYOSHI, Y. & UEDA, R. (1991). The p53 gene is very frequently mutated in small-cell lung cancer with a distinct nucleotide substitution pattern. *Oncogene*, **6**, 1775-1778.

VOJTESEK, B., BARTEK, J., MIDGLEY, C.A. & LANE, D.P. (1992). An immunochemical analysis of the human nuclear phosphoprotein-p53 - new monoclonal antibodies and epitope mapping using recombinant-p53. *J. Immunol. Methods*, **151**, 237-244.

WADE-EVANS, A. & JENKINS, J.R. (1985). Precise epitope mapping of the murine transformation-associated protein. p53. *EMBO J.*, **46**, 575-583.

WINTER, S.F., MINNA, J.D., JOHNSON, B.E., TAKAHASHI, T., GAZDAR, A.F. & CARBONE, D.P. (1992). Development of antibodies against p53 in lung cancer patients appears to be dependent on the type of p53 mutation. *Cancer Res.*, **52**, 4168-4174.